# Enhancing Medical Image Classification through Transfer Learning and CLAHE Optimization

**DOI:** 10.2174/0115734056342623241119061744

**Published:** 2025-01-17

**Authors:** Kamal Halloum, Hamid Ez-Zahraouy

**Affiliations:** 1 Laboratory of Condensed Matter and Interdisciplinary Sciences, CNRST Labeled Research Unit, URL-CNRST, Faculty of Sciences, Mohammed V University in Rabat, Rabat, Morocco

**Keywords:** Medical image classification, CLAHE, Transfer learning, Data augmentation, Diagnostic accuracy, Tumor

## Abstract

**Introduction:**

This paper examines the impact of transfer learning and CLAHE (Contrast Limited Adaptive Histogram Equalization) optimization on the classification of medical images, specifically brain images.

**Methods:**

Four different setups were tested: normal images without data augmentation, normal images with data augmentation, CLAHE-processed images without data augmentation, and CLAHE-processed images with data augmentation.

**Results:**

The results show that using CLAHE combined with data augmentation significantly improves classification accuracy. Specifically, the combination of CLAHE and data augmentation achieved a precision of 0.90, a recall of 0.87, an F1-score of 0.89, and an accuracy of 0.86, outperforming the other setups.

**Conclusion:**

These findings highlight the effectiveness of CLAHE optimization in the context of transfer learning, particularly when data augmentation techniques are also applied, leading to an overall improvement in the performance of brain image classification models.

## 1 INTRODUCTION

Medical image classification plays a critical role in the diagnosis and treatment planning. Automated methods have been developed to provide accurate, objective, and fast classifications in various medical departments, particularly in pathology and dermatology, where diagnosis often relies on visual examinations of skin lesions. Recent advancements in image processing techniques, such as CLAHE, have shown promise in enhancing image quality by improving local contrast and detail visibility [1, 2]. Concurrently, Transfer Learning has emerged as a powerful approach in machine learning, allowing pretrained models to be adapted for new tasks with limited data. This study explores the combined use of CLAHE for image enhancement and Transfer Learning for feature extraction in the context of medical image classification [3]. The objective is to investigate how these techniques can synergistically improve classification accuracy, particularly in scenarios where image quality and data augmentation strategies significantly influence diagnostic outcomes [4]. This research aims to contribute insights into optimizing medical image analysis, thereby potentially enhancing diagnostic accuracy and improving patient care outcomes [5].

Despite these advancements, the field still faces significant challenges. One of the primary obstacles is the scarcity of labeled medical image data, which is crucial for training robust machine learning models [6]. Additionally, the quality of medical images can vary widely due to factors such as noise, low contrast, and imaging artifacts, further complicating the classification process. Addressing these challenges is essential for improving the performance and reliability of automated classification systems [7].

This study explores the combined use of CLAHE for image enhancement and Transfer Learning for feature extraction in the context of medical image classification [8]. The objective is to investigate how these techniques can synergistically improve classification accuracy, particularly in scenarios where image quality and data augmentation strategies significantly influence diagnostic outcomes [9]. This research aims to contribute insights into optimizing medical image analysis, thereby potentially enhancing diagnostic accuracy and improving patient care outcomes [10].

Recent literature highlights various approaches to overcoming these challenges. For instance, a deep learning framework was developed to predict the severity of COVID-19 infection, showcasing the potential of advanced machine learning techniques in medical image analysis. Additionally, interpretability in neural networks has become a critical area of focus, as demonstrated by studies on real-time interpretable repairs of deep neural classifiers. The importance of high-quality imaging data is further emphasized in research on hemodynamic characteristics in microvascular structures using high-fidelity red blood cell simulations [11]. Moreover, novel techniques like sparse coding have been proposed to enhance hyperspectral medical image data augmentation, addressing the issue of data scarcity [12]. In the context of therapeutic applications, machine learning models have been employed to predict anticancer peptide activity, demonstrating the versatility of these techniques in various medical domains [13].

In today's age of technology, machine learning plays an essential role in the medical field, particularly in oncology. It is particularly useful for solving problems such as medical image classification, tumor detection [14], as well as organ segmentation and the detection of suspicious anomalies [15]. The CNNs are widely recognized as one of the most effective techniques in machine learning, particularly for classification. However, despite steady progress in these techniques, results have yet to live up to expectations due to various challenges, including data imbalance and lack of data. In addition, the lack of specific data pre-processing hampers the ability of neural networks to discern imported data.

For these reasons, our study aims to discover effective techniques for improving image contrast and thus increasing model accuracy. In this context, several studies have been carried out using the CLAHE technique [16]. For example, a study of brain tumor classification [17] focused solely on the use of the HE technique for image preprocessing, achieving acceptable accuracy. However, the use of the CLAHE technique could potentially significantly improve results. Another comparative study [18] demonstrated that using the CLAHE technique for image preprocessing, accuracy reached 82.3%, compared to 79% without preprocessing. In addition, a separate study showed that the CLAHE technique greatly contributed to the detection of simulated speculations in dense mammograms by improving image contrast [19].

In addition, we identified a particularly relevant study that combined two techniques, namely CLAHE and the application of a Gaussian filter, to preprocess a set of images. This study demonstrated a significant improvement in accuracy, reaching an impressive 98.75% over non-preprocessed images [20]. Furthermore, a fascinating study was conducted to compare two commonly used preprocessing methods, namely CLAHE and Discrete Cosine Transform (DCT), in the context of CNN assisted diagnosis using a mixed, open-source dataset. The results of this experimental study strongly suggested that the DCT method outperformed the CLAHE method in terms of accuracy on the test set [21].

In addition, we found a study similar to ours focusing on the classification of chest X-ray images using the Resnet-101 neural network. This study provided a relevant comparison of accuracy between CLAHE-preprocessed and non-preprocessed data. Results for normative parameters, including accuracy, sensitivity and specificity, were 99.22%, 99.58% and 98.91% for the original data, and 99.91%, 99.62% and 99.60% for CLAHE-pretreated data [22].

In this paper, we make several important contributions. First, we show how using both CLAHE and Transfer Learning together can improve the quality of medical images by enhancing contrast and detail. Second, we examine how these techniques can boost classification accuracy, especially in cases where image quality and data augmentation are key factors. Third, we compare different methods for preprocessing images and highlight the benefits of using CLAHE. Finally, we discuss how our findings can help improve the analysis of medical images, potentially leading to better diagnostic accuracy and patient care. In Section 2, we will cover the image processing techniques and Transfer Learning. Section 3 describes our experimental study, including the data and methods we used. Section 4 provides a comparison of the results, and Section 5 concludes the paper by summarizing our main findings and suggesting future research directions.

### 1.1 Objectify

The objective of this work is to enhance the accuracy of medical image classification, particularly in brain MRI images, by leveraging the CLAHE technique for image preprocessing and Transfer Learning for feature extraction. By systematically analyzing the impact of these methods, both with and without Data Augmentation, we aim to demonstrate how these approaches can improve contrast and detail in medical images, leading to more reliable classification results and potentially contributing to better diagnostic outcomes.

## 2 METHOD

In order to obtain accurate results, we chose to carry out a classification of brain images, distinguishing between healthy and cancer-affected brains. This classification was carried out in two distinct modes: firstly, without the use of Data Augmentation, and then in a second mode, using a Data Augmentation approach. The latter approach included the integration of several parameters aimed at increasing the number of imported images.

The fundamental objective of this process was to thoroughly analyze the impact of the CLAHE technique on improving the accuracy of a model based on CNNs.

Our study was divided into different, well-detailed sections, and the flowchart in Fig. (**1**) details the successive stages of the process we adopted. This process included the following phases: data acquisition, conventional preprocessing, pre-processing using the CLAHE technique, data segmentation, and finally, classification of the brain MRI images. Each of these steps will be explained in detail below.

### 2.1 Data Acquisition

To implement our study, we selected a set of brain MRI images from the Kaggle dataset “Brain MRI Images for Detecting Brain Tumors,” as described in reference [23]. This dataset includes 253 images, categorized into two groups: 155 images depict normal brain structures without tumors (non-tumorous), and 98 images represent cases of brain tumors (tumorous). The images primarily capture axial views of the brain, focusing on the detection of gliomas, a common type of brain tumor. These MRI scans, acquired in T1-weighted modality, are known for providing detailed anatomical information, making them particularly useful for identifying and analyzing brain lesions.

**Fig. (2):**
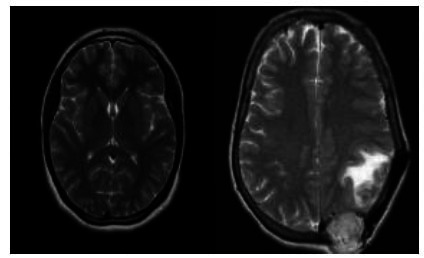
(Left) A normal brain, (Right) A brain affected by a tumor.

Before applying the CLAHE technique, the images underwent several preprocessing steps to ensure consistency and enhance the quality of the input data. First, all images were resized to a uniform dimension to maintain consistency in the input to our model. Next, image normalization was performed to standardize the pixel intensity values across the dataset, reducing variability caused by different imaging conditions. Finally, any noise present in the images was minimized using a Gaussian filter, which helped to preserve the structural details essential for accurate classification. Although the dataset's size may seem limited, our goal is to investigate a technique that can achieve high accuracy even with a smaller number of images. (Fig. **2**).

### 2.2 Classic Pre-treatment

Conventional data augmentation methods are commonly used when deploying deep learning models, especially when the amount of data available is limited [24]. In this study, we implemented various augmentation strategies to enhance the diversity and volume of our image dataset, thus addressing the challenges of limited data [25]. The augmentation parameters are summarized in Tables **1**-**2**. These techniques include rotations, scaling, translations, and flips, all aimed at artificially increasing the dataset size [26]. By introducing variability in the training data, we aimed to improve the model's robustness and generalizability. Augmentation helps prevent overfitting and enables the model to learn invariant features from the images, ultimately leading to better performance on unseen data. Additionally, the use of CLAHE for image enhancement further improved the quality of the images, ensuring that critical features were preserved and emphasized during the augmentation process [27]. This dual approach, combining CLAHE with conventional Data Augmentation techniques, provides a comprehensive strategy to tackle the issue of insufficient data and enhance the accuracy of medical image classification [28].

#### 2.2.1 Data Augmentation

Data augmentation is crucial when using deep learning models, especially when data is limited [29]. In this study, we used various augmentation techniques to increase the diversity and size of our image dataset, addressing the problem of having too little data [30]. The augmentation methods are listed in Table **2**. These techniques include rotations, scaling, translations, and flips, all designed to artificially enlarge the dataset [31]. By adding variability to the training data, we aimed to make the model more robust and better at generalizing.

Data augmentation helps prevent overfitting and allows the model to learn invariant features from the images, which leads to better performance on new, unseen data. This idea is supported by recent research, such as the study on GAN-based augmentation using a hybrid loss function for dermoscopy images [32], 2024. Additionally, using CLAHE for image enhancement improved image quality by preserving and highlighting important features during augmentation [33]. Combining CLAHE with conventional data augmentation techniques provides a comprehensive approach to tackle the issue of insufficient data and enhance medical image classification accuracy [34].

### 2.3 Image Pre-processing

The quality of resulting Magnetic Resonance Images (MRI) is often degraded by factors such as Gaussian and salt-and-pepper noise [35]. This degradation complicates a neural network's ability to identify all the features present in the image. MRI image denoising enables corrupted images to be converted into high-quality images, which can then be used in a variety of machine learning applications. There are many methods for denoising MRI images, each with its own challenges and advantages.

In this context, we propose the use of a widely recognized technique for improving the contrast of MRI images, namely the CLAHE method, which we integrate into the training process. CLAHE is known for its ability to enhance fine details while preserving edges, which is crucial for accurate medical image analysis. By applying CLAHE, we aim to improve the quality of the input images, making it easier for the neural network to extract relevant discriminative features. This integration aims to optimize the model's performance by increasing the clarity and distinction of structures in MRI images, thereby contributing to more precise and reliable classification.

#### 2.3.1 Contrast Limited Adaptative Histogram Equalization (CLAHE)

Originally designed to improve low-contrast images, it is also used to solve the problem of noise amplification caused by the application of the Histogram Equalization (HE) technique. Specifically, it divides the image into small regions called blocks, generally 8x8 in size, in order to equalize the histogram of each region individually [36].

However, the CLAHE technique has two essential parameters: the Block Size (BS) and the Clipping Limit (CL), which play a central role in enhanced image quality [37]. Indeed, when the Clipping Limit (CL) is increased, the image becomes brighter. This is because the original image has a very low intensity with a higher CL value, resulting in a flatter histogram [38]. By increasing the clipping limit, the dynamic range of the image expands, leading to an increase in image contrast [39]. Fig. (**3**) shows how the CLAHE technique affects medical images.

### 2.4 Deep CNN Architecture

CNNs are seen as an effective solution to problems associated with medical image processing. Thanks to their advanced functionalities and their ability to process three-dimensional images, they enable rapid classification, particularly of infectious or tumoral cases, with a high degree of certainty. What's more, they can be used to distinguish between similar conditions [40].

The structure of a CNN is based on three fundamental layers: the convolution layers use a filter (the kernel) with dimensions strictly smaller than those of the input image, which efficiently convolves the image matrix to extract all relevant information. The Pooling Layers are then responsible for reducing the dimensions, parameters and features of the image, cutting them in half, thus facilitating network training. Finally, Fully Connected Layers constitute the last layer, where all the mathematical operations required to produce the desired results are performed [41].

Building a comprehensive CNN for a medical image dataset remains a complex challenge, particularly with regard to model quality and accuracy. However, transfer learning can solve this problem. With this in mind, we chose the VGG-16 architecture because of its ability to enable fine-tuning, which allows us to modify the parameters of a pre-trained CNN model, freezing some layers and refining other.

### 2.5 Hyperparameter

This study adjusted several critical hyperparameters for classification simulations. We conducted multiple experiments by varying these parameters to determine which configurations would achieve high accuracy levels. Ultimately, we identified the most influential values that enabled the algorithm to achieve superior accuracy results, as depicted in Table **1**.

The adjusted hyperparameters included batch size, learning rate, number of epochs, and regularization parameters. Each parameter was tested individually and in combinations to assess their impact on model performance. The experimental outcomes demonstrated that precise adjustments of these parameters significantly enhanced classification accuracy. For instance, appropriate learning rates were crucial to mitigate issues of overfitting or underfitting, while optimal batch sizes contributed to improved model convergence.

Furthermore, the effects of regularization techniques, such as L2 regularization and dropout, were studied to prevent overfitting and enhance model generalization on unseen data. The selected final parameters were those that yielded the best overall performance in terms of accuracy and model stability. These findings underscore the importance of meticulous hyperparameter optimization to maximize the capabilities of classification models in medical imaging applications.

## 3 EXPERIMENTAL

### 3.1 Implementation Google Colab

In our study, we conducted multiple simulations for brain image classification. A total of 40 simulation experiments were carried out to achieve high accuracy and convergence. These experiments were divided into two sets: 20 focused on classification without Data Augmentation, and the remaining 20 on classification with Data Augmentation. It's important to note that running these experiments required significant time and high computational resources. Therefore, we opted to use Google Colab, which provides a faster GPU execution environment to expedite the process.

During the simulations without Data Augmentation, we examined how the model performed using only the original data without any additional modifications to increase dataset size or variability. Conversely, experiments with data augmentation explored the impact of techniques such as rotation, resizing, and flipping on the model's ability to generalize and improve accuracy in brain image classification.

Each experiment was carefully planned to ensure consistent conditions and reliable results. Choosing Google Colab as our execution platform was crucial in optimizing simulation efficiency by leveraging available GPU resources, thereby significantly reducing computation time compared to traditional computing environments. This approach enabled us to conduct our simulations within reasonable timeframes while ensuring thorough analysis of the brain image classification model's performance.

### 3.2 Performance Metrics

Performance metrics are essential for evaluating the effectiveness of a classification model. In our study, we used several key indicators to measure the performance of our brain image classification model. These metrics included accuracy, which represents the percentage of correct predictions out of all predictions made. We also considered recall and specificity, which measure the model's ability to correctly identify positive and negative cases, respectively. Additionally, we used the ROC (Receiver Operating Characteristic) curve and the AUC (Area Under the Curve) to assess the overall performance of the model at different classification thresholds. These metrics provide a comprehensive view of the model's ability to distinguish between classes, accounting for potential data imbalances. By combining these different indicators, we obtained a precise and thorough evaluation of the performance of our brain image classification model.

#### 3.2.1 Accuracy

Accuracy is of crucial importance when evaluating and refining a model or architecture, as it measures the ability to perform classification correctly. The corresponding formula is illustrated in the following (Eq. **1**):


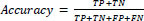


#### 3.2.2 Precision

This is a parameter used to evaluate the ability of a model or architecture to correctly classify positive cases (yes/yes) (Eq. **2**).


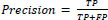


#### 3.2.3 Sensitivity (Recall)

Sensitivity, also known as Recall, is a parameter used to evaluate the ability of a model or architecture to correctly classify all positive and negative cases (Eq. **3**).


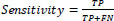


#### 3.2.4 Specificity

This is noted as the rate of true negatives, and is used to assess the precision of data classified as negative correctly or appropriately identified (Eq. **4**).


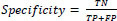


#### 3.2.5 Score

This is an essential parameter which is used to evaluate the overall effectiveness of two other parameters: accuracy and precision. Its range of values generally varies between 0 and 1 (Eq. **5**).


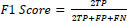


## 4 RESULTS

**Fig. (4):**
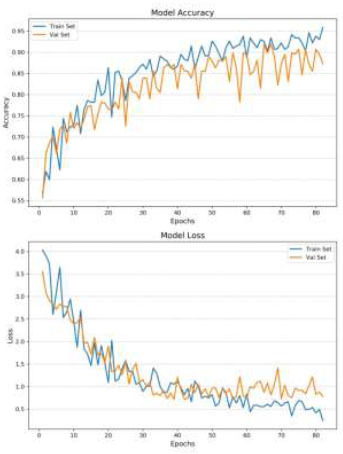
Loss and accuracy - CLAHE images, with data augmentation.

**Fig. (5):**
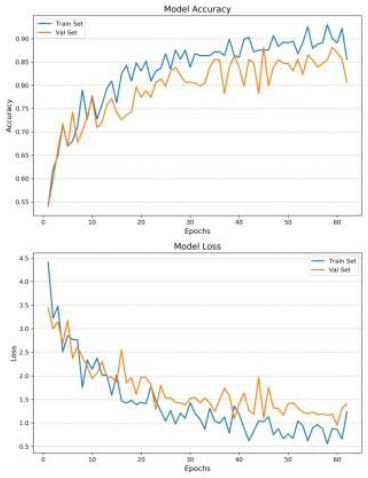
Loss and accuracy - CLAHE images, no data augmentation.

The CLAHE technique has a significant impact on the classification accuracy of brain medical images. The results show that CLAHE-enhanced images consistently outperform normal images across all simulations. Specifically, when data augmentation is applied to CLAHE-enhanced images (Fig. **4**), the model achieved an accuracy of 0.86 after 80 epochs. Even without data augmentation, CLAHE-enhanced images reached an accuracy of 0.80 in 50 epochs (Fig. **5**). This improvement indicates that CLAHE effectively enhances image contrast, which allows the VGG-16 model to extract more discriminative features from the images. The superior performance of CLAHE-enhanced images underscores the importance of image preprocessing in improving the accuracy of deep learning models in medical image classification.

**Fig. (6):**
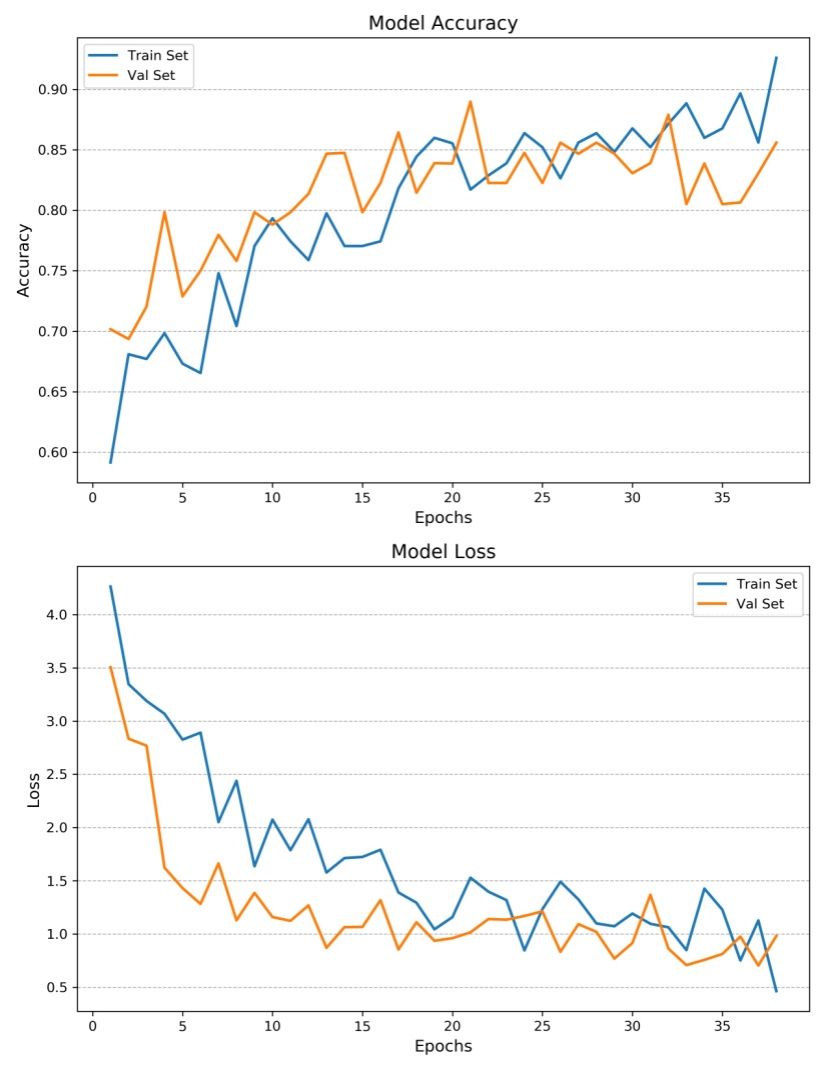
Loss and accuracy - normal images, with data augmentation.

**Fig. (7):**
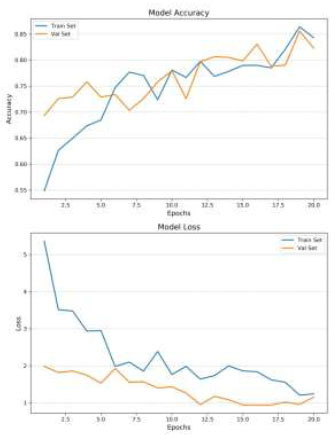
Loss and accuracy - normal images, no data augmentation.

Data augmentation plays a crucial role in enhancing the model's performance by introducing variability into the training set, thereby reducing overfitting. In the case of normal images, data augmentation increased the model’s accuracy from 0.78 to 0.84 and significantly reduced overfitting (Table **3**), allowing the model to generalize better to unseen data (Figs. **6** and **7**). For CLAHE-enhanced images, the combination with data augmentation resulted in the highest accuracy of 0.86, demonstrating the synergistic effect of these two techniques. This finding highlights the effectiveness of data augmentation in conjunction with CLAHE, as it not only improves classification accuracy but also enhances the model’s robustness by exposing it to a broader range of variations during training.

The analysis also reveals the impact of overfitting and the efficiency of training under different conditions. When the model was trained on normal images without Data Augmentation (Fig. **6**), it quickly reached an accuracy of 0.84 within 20 epochs, but this came with significant overfitting. The model’s inability to generalize well in this case indicates that the training data lacked sufficient variability, causing the model to perform poorly on the validation set. In contrast, the introduction of Data Augmentation mitigated overfitting and increased accuracy, particularly for normal images. However, the best balance between accuracy and generalization was achieved with CLAHE-enhanced images, which benefited from both improved image quality and the regularizing effect of data augmentation.

The number of training epochs required for convergence varied across the simulations, reflecting the complexity of the learning task under different conditions. The model trained on CLAHE-enhanced images with data augmentation required 80 epochs to achieve the highest accuracy of 0.86. This longer training duration indicates that while the model took more time to converge, it ultimately learned more robust and discriminative features, leading to superior classification performance. In contrast, the model trained on normal images without data augmentation converged quickly, reaching 0.84 accuracy in just 20 epochs, but this came at the cost of poorer generalization and significant overfitting. The difference in training efficiency highlights the trade-off between rapid convergence and the quality of the learned model, with CLAHE-enhanced images and Data Augmentation providing the best results.

**Fig. (8):**
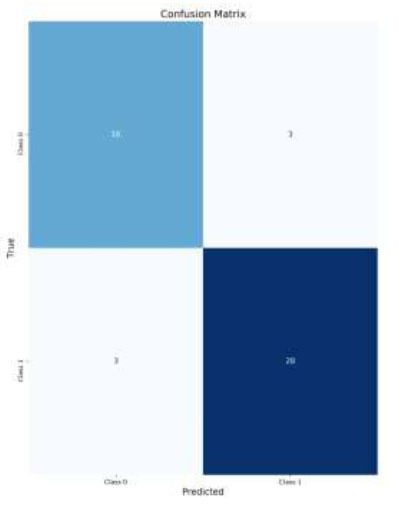
Confusion matrix for CLAHE images with data augmentation.

**Fig. (9):**
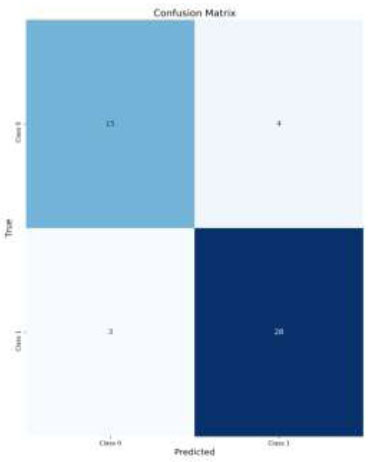
Confusion matrix for CLAHE images without data augmentation.

**Fig. (10):**
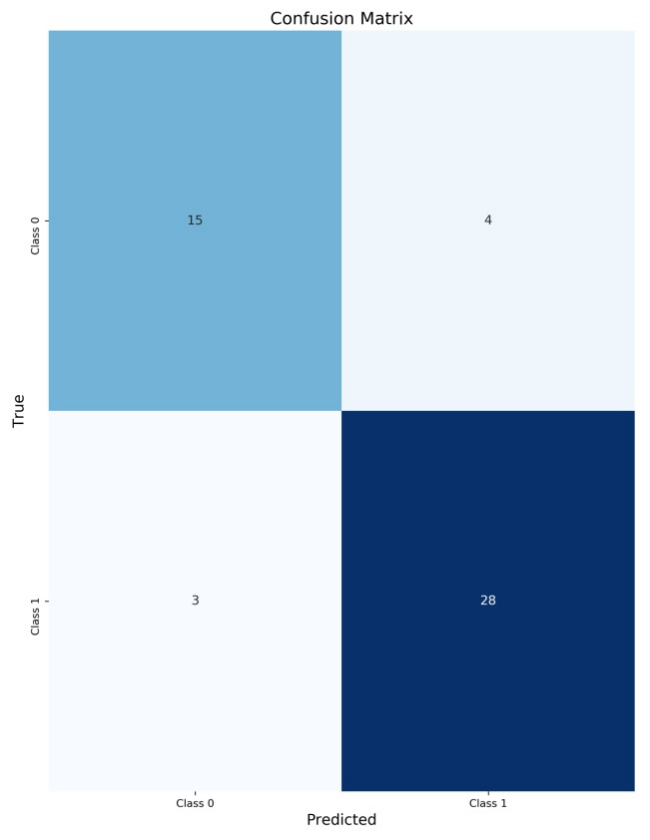
Confusion matrix for normal images with data augmentation.

**Fig. (11):**
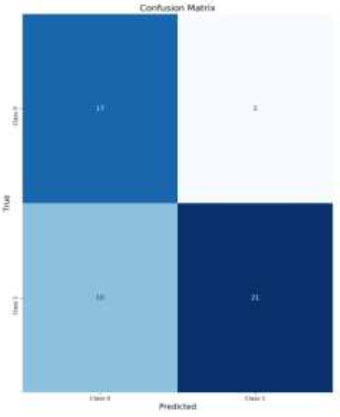
Confusion matrix for normal images without data augmentation.

The analysis of the classification performance for brain medical images reveals the effectiveness of applying CLAHE and Data Augmentation techniques. For normal images without Data Augmentation (Figs. **8**-**11***),* the model achieved an accuracy of 84%, indicating a good balance between True Positives (TP) and True Negatives (TN). Introducing Data Augmentation for normal images decreased accuracy to 78%, suggesting that while Data Augmentation may have introduced variability, it led to fewer False Positives (FP) but also more False Negatives (FN) (Fig. **8**).

When CLAHE was applied without Data Augmentation, the accuracy was 80% (Fig. **9**). This result is slightly lower than the 84% accuracy achieved with normal images without Data Augmentation, but still demonstrates effective classification. CLAHE improves contrast, which benefits classification, though not as much as when combined with Data Augmentation.

The combination of CLAHE with Data Augmentation achieved the highest accuracy at 86% (Fig. **10**). This indicates that the integration of CLAHE with Data Augmentation significantly enhances the model's performance, reducing the number of false positives and false negatives while improving the model’s ability to correctly classify images. This suggests that both CLAHE and Data Augmentation complement each other well, providing a robust solution for improving classification accuracy in medical imaging.

## 5 DISCUSSION

The combination of CLAHE enhancement and data augmentation consistently produces the most accurate and balanced classification results, as evidenced by the lowest false positive and false negative rates. This combination allows the model to generalize effectively across varying data conditions, leading to a significant improvement in classification performance. In contrast, models trained without data augmentation or without CLAHE enhancement exhibit higher misclassification rates, highlighting the importance of these techniques in medical image analysis.

## CONCLUSION

In our study aimed at improving the accuracy of medical image classification using CNNs, we found that conventional image preprocessing often falls short in achieving satisfactory accuracy. However, employing the CLAHE technique proved effective by enhancing the contrast of medical images, significantly boosting model accuracy.

Our findings clearly demonstrate that CLAHE not only enhances the visual quality of images by improving contrast and revealing finer details but also strengthens the model's ability to extract crucial features essential for precise classification. Models trained on CLAHE-preprocessed images consistently outperformed those using non-preprocessed images, as evidenced by our experiments.

In conclusion, integrating CLAHE into medical image preprocessing represents a significant advancement in improving CNN accuracy for medical image classification. This promising approach holds potential for more accurate applications in medical imaging, with substantial implications for clinical diagnosis and patient management.

## Figures and Tables

**Fig. (1):**
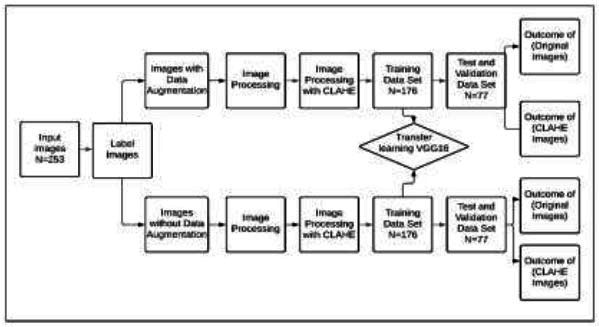
Diagram illustrating all the stages in the classification process.

**Fig. (3):**
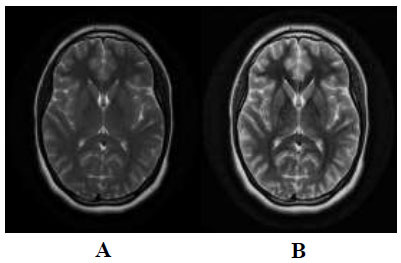
(**a**) Original image, (**b**) Image enhanced with CLAHE.

**Table 1:** Hyperparameters used in the classification model.

**Data**	**Batch Size**	**Nb of Iteration**	**Nb of Epoches**	**Type of Optimizer**	**Early Stopping**	**Type of metrics**
Train:70% Val: 10% Test: 20%	Train: 16 Val: 4 Test: 8	11	100	Adam (0.0001)	10	Accuracy

**Table 2:** Parameter configuration for traditional data augmentation method.

**Method**	**Setting**
Rotation	40°
Shearing	0.2
Image zoom	0.2
Image height range	0.2
Image width range	0.2
Image horizontal	True
Image vertical	True
Fill mode	'nearest'

**Table 3:** Performance metrics for brain image classification methods.

**Cas**	**Precision**	**Recall**	**F1 - score**	**Accuracy**
Normal images, no data augmentation	0.87	0.87	0.87	0.84
Normal images, with data augmentation	0.88	0.74	0.81	0.78
CLAHE images, no data augmentation	0.84	0.84	0.84	0.80
CLAHE images, with data augmentation	0.90	0.87	0.89	0.86

## Data Availability

The data and supportive information is available within the article.

## LIST OF ABBREVIATIONS

CLAHE: Contrast Limited Adaptive Histogram Equalization
DCT: Discrete Cosine Transform
MRI: Magnetic Resonance Images
HE: Histogram Equalization
CL: Clipping Limit
BS: Block Size
ROC: Receiver Operating Characteristic
AUC: Area Under the Curve

## References

[1] Suzuki K. (2017). Overview of deep learning in medical imaging.. Radiological Phys. Technol..

[2] Khan M.A.A., Khan M.S.A.P., Ali A.F.S.U., Alzubaidi A. (2022). A review on deep learning in medical image analysis.. J. King Saud Uni. Comput. Inform. Sci..

[3] Raghu M., Zhang C., Kleinberg J., Bengio S. (2019). Transfusion: Understanding transfer learning for medical imaging.. Adv. Neural Inform. Process. Sys..

[4] Pan S.J., Yang Q. (2010). A survey on transfer learning.. IEEE Trans. Knowl. Data Eng..

[5] Zhou Y., Wang Y., Zhou Y., Jiang Y., He L. (2020). Classification of medical images based on deep learning with adaptive histogram equalization and image augmentation.. Comput. Math. Methods Med..

[6] Li Z., Zhao S., Chen Y., Luo F., Kang Z., Cai S., Zhao W., Liu J., Zhao D., Li Y. (2021). A deep-learning-based framework for severity assessment of COVID-19 with CT images.. Expert Syst. Appl..

[7] Catak F.O., Yue T., Ali S. (2022). Uncertainty-aware prediction validator in deep learning models for cyber-physical system data.. ACM Trans. Softw. Eng. Methodol..

[8] Hee E. (2022). Transfer learning for medical image classification: A literature review.. BMC Med. Imag..

[9] Quadri R., Deshpande A. (2022). Deep learning-based segmentation and classification of COVID-19 infection severity levels from CT scans.. Revue d’Intelligence Artificielle.

[10] Taouli A., Bensaber D.A., Bencherif K., Keskes N. (2022). Semantics convolutional neural network for medical images analysis.. Revue d’Intelligence Artificielle.

[11] Hossain M.M.N., Hu N.W., Kazempour A., Murfee W.L., Balogh P. (2024). Hemodynamic characteristics of a tortuous microvessel using high fidelity red blood cell resolved simulations.. Microcirculation.

[12] Nalepa J., Myller M., Kawulok M. (2019). Hyperspectral data augmentation.. arXiv.

[13] Basith S., Manavalan B., Shin T.H., Lee D.Y., Lee G. (2020). Evolution of machine learning algorithms in the prediction and design of anticancer peptides.. Curr. Protein Pept. Sci..

[14] LeCun Y. (2018). The power and limits of deep learning.. Res. Technol. Manag..

[15] Sundaram M., Ramar K., Arumugam N., Prabin G. (2011). Histogram modified local contrast enhancement for mammography images.. Appl. Soft Comput..

[16] Lu H-C. The Classification of mammogram using convolutional neural network with specific image preprocessing for breast cancer detection.. 2019 2nd International Conference on Artificial Intelligence and Big Data (ICAIBD).

[17] Pisano E.D., Zong S., Hemminger B.M., DeLuca M., Johnston R.E., Muller K., Braeuning M.P., Pizer S.M. (1998). Contrast limited adaptive histogram equalization image processing to improve the detection of simulated spiculations in dense mammograms.. Digital Imag. J..

[18] Kusrini K., Muhammad R.A.Y., Al Fatta H. (2022). The effect of gaussian filter and data preprocessing on punakawan puppet image classification with convolutional neural network algorithm.. Iran. J. Electr. Comput. Eng..

[19] Cui Y., Ding J., Liu M., Liu X. CNN-based COVID-19 pneumonia diagnosis: A comparative study on different image preprocessing method.. 2021 2nd International Seminar on Artificial Intelligence, Networking and Information Technology (AINIT).

[20] Yasarla K.M., Krishna M.S., Dhanunjaya B. (2020). Improving accuracy of mammogram classification using CLAHE and Gaussian filter.. Int. J. Comput. Appl..

[21] Srivastava R., Singh A. (2021). Enhancing the performance of deep learning models for brain tumor classification using combined CLAHE and Gaussian filter.. J. Digit. Imaging.

[22] Gupta R., Chatterjee S. (2020). Comparative analysis of preprocessing techniques for CNN-based diagnosis: CLAHE *vs*. Discrete Cosine Transform.. IEEE Access.

[23] Chakrabarty N. Brain MRI images for brain tumor detection.. https://www.kaggle.com/datasets/navoneel/brain-mri-images-for-brain-tumor-detection.

[24] Fabbri A., Furlan A., Masciocchi C., Gatti M. (2020). Data augmentation strategies for deep learning in medical image analysis.. Med. Image Anal..

[25] Perez L., Wang J. (2017). The effectiveness of data augmentation in image classification using deep learning.. arXiv.

[26] Auguste K.A., Wong W.Y., Cavanagh P. (2022). Data augmentation techniques in medical image analysis: A comprehensive review.. Med. Image Anal..

[27] Ronneberger O., Fischer P., Brox T. (2015). U-Net: Convolutional networks for biomedical image segmentation.. Medical Image Computing and Computer-Assisted Intervention – MICCAI 2015.

[28] Wang S., Summers R.M. (2012). Machine learning and radiology.. Med. Image Anal..

[29] Shorten C., Khoshgoftaar T.M. (2019). A survey on image data augmentation for deep learning.. J. Big Data.

[30] Zhang J. (2020). Data augmentation for medical imaging: A review.. Med. Image Anal..

[31] Taylor L., Nitschke G. (2018). Improving deep learning with generic data augmentation techniques.. IEEE Comput. Graph. Appl..

[32] Frid-Adar M., Klang E., Amitai M., Goldberger J., Greenspan H. Synthetic data augmentation using GAN for improved liver lesion classification.. 2018 IEEE 15th International Symposium on Biomedical Imaging (ISBI 2018).

[33] Zhou Z., Siddiquee M.M.R., Tajbakhsh N., Liang J. (2020). UNet++: Redesigning skip connections to exploit multiscale features in image segmentation.. IEEE Trans. Med. Imaging.

[34] Kumar R., Gupta P. (2021). A comprehensive survey on denoising techniques for MRI images.. Med. Image Anal..

[35] Shah D.O., Hing F.N., Phatak S.V., Mavroforakis C. (2020). A comprehensive review of denoising techniques for MRI images.. J. Imaging Sci. Technol..

[36] Pizer S.M., Amburn E.P., Austin J.D., Cromartie R., Geselowitz A., Greer T., ter Haar Romeny B., Zimmerman J.B., Zuiderveld K. (1987). Adaptive histogram equalization and its variations.. Comput. Vis. Graph. Image Process..

[37] Zuiderveld K. (1994). Contrast limited adaptive histogram equalization.

[38] Mokoena P., Pheeha K. (2020). A comparative study of CLAHE and histogram equalization for contrast enhancement in digital images.. Advances in Science Technology and Engineering Systems Journal.

[39] Reza A.M. (2004). Realization of the contrast limited adaptive histogram equalization (CLAHE) for real-time image enhancement.. J. VLSI Signal Process..

[40] Litjens G., Kooi T., Bejnordi B.E., Setio A.R., Ciompi F., Ghafoorian M., van Ginneken B. (2017). A survey on deep learning in medical image analysis.. Med. Image Anal..

[41] Simonyan K., Zisserman A. (2015). Very deep convolutional networks for large-scale image recognition.. arXiv:1409.1556.

